# Pulse-Generation Maps in External-Cavity Semiconductor Lasers with Short-Delay Optical Feedback

**DOI:** 10.3390/s26206404

**Published:** 2026-10-10

**Authors:** Gerardo Antonio Castañón Ávila, Ana Maria Sarmiento-Moncada, Alejandro Aragón-Zavala, Ivan Aldaya Garde

**Affiliations:** 1 School of Engineering and Science, Tecnologico de Monterrey, Ave. Eugenio Garza Sada 2501, Monterrey 64849, Nuevo Leon, Mexico; anamaria.sarmiento@tec.mx; 2 Universidad Autonoma de Nuevo Leon (UANL), San Nicolas de los Garza 66455, Nuevo Leon, Mexico; 3 Department of Electronics and Mechatronics, Tecnologico de Monterrey, Queretaro Campus, Queretaro 76130, Queretaro, Mexico; aaragon@tec.mx; 4 School of Engineering of Sao Joao da Boa Vista, Sao Paulo State University (UNESP), Sao Joao da Boa Vista CEP 13876-750, SP, Brazil; ivan.aldaya@unesp.br

**Keywords:** pulse maps, integrated photonics, external-cavity semiconductor laser, nonlinear photonics

## Abstract

We investigate pulse generation in external-cavity semiconductor lasers subject to short-delay optical self-feedback using a deterministic quadrature-based delay-differential model. High-resolution pulse-generation maps are constructed in the $\left(\mathrm{lg}\left(C\right),\phi_{f}\right)$ parameter space, where $\mathrm{lg}\left(C\right)\equiv \log_{10}\left(C\right)$, for pump ratios μ=4, 6, 8, and 10, where $\mu =J/J_{\mathrm{th}}$. For each pump level, the feedback delay is normalized to the solitary-laser relaxation-oscillation period and set to $\tau_{f}/T_{R}=0.5$ and 1, providing a physically consistent basis for comparing pulsing regimes across operating conditions. Post-transient intensity traces are classified in the time domain using complementary measures of pulse separation, temporal regularity, pulse width, duty factor, valley-to-peak ratio, peak prominence, baseline occupancy, and autocorrelation, thereby reducing the misclassification of strongly distorted periodic oscillations as regular pulse trains. The resulting maps characterize the existence, repetition rate, mean pulse width, and duty factor of the detected pulses. The results reveal that pulse generation is governed by the combined effects of feedback strength, feedback phase, pump ratio, and normalized delay. Notably, increasing the pump ratio systematically shifts the pulsing regimes toward higher repetition rates, with representative values increasing from approximately 2 GHz at μ=4 to above 7 GHz at μ=10, demonstrating clear pump-controlled tuning of the generated pulse trains. The considered short delays correspond to millimeter- to centimeter-scale optical feedback paths for representative photonic platforms. These results provide a reproducible framework for identifying feedback-induced regular pulsing regimes and may support the design of compact semiconductor-laser sources for sensing, ranging, and integrated photonic applications.

## 1. Introduction

Compact optical pulse sources are of particular interest for sensing and ranging systems, where accurately timed optical pulses can provide direct temporal information about propagation, reflection, and scattering processes. In pulsed light detection and ranging (LiDAR) and time-of-flight measurements, the pulse duration and repetition rate influence the attainable temporal resolution, measurement rate, and ranging ambiguity [1,2,3,4]. Related time-domain interrogation principles are also widely employed in optical time-domain reflectometry and distributed fiber sensing [5,6,7]. The continuing development of solid-state and photonic-integrated LiDAR further motivates compact optical sources whose temporal characteristics can be controlled without complex external modulation architectures [8,9,10,11,12,13].

Stable optical pulse trains are also relevant to high-speed communications, microwave photonics, signal processing, and neuromorphic photonics. Periodic pulses can be used for optical timing, clock recovery, radio-frequency and microwave signal generation, and event-based photonic processing [14,15,16,17]. Operation near 1550nm is particularly attractive because it coincides with the low-loss transmission window of standard optical fibers and with mature semiconductor and integrated-photonic technologies [18,19]. Consequently, the ability to generate and tune regular pulse trains using compact semiconductor-laser configurations is relevant to both sensing-oriented and communication-oriented photonic systems [20].

Several mechanisms have been employed to generate optical pulses in semiconductor lasers, including gain switching, direct current modulation, mode locking, saturable absorption, optical injection, optoelectronic feedback, and delayed optical self-feedback [21,22]. Gain-switched and actively modulated lasers require an external electrical drive, while passively mode-locked devices generally require additional cavity sections or saturable absorbers. Optical injection provides substantial dynamical control but requires an independent master laser and accurate adjustment of the injection strength and frequency detuning. Delayed optical self-feedback provides a comparatively simple alternative in which a fraction of the emitted optical field is returned to the laser after propagation through an external path, allowing the laser dynamics to be controlled without an independent optical source.

Pulse generation induced or controlled by delayed optical feedback has been reported in several semiconductor-laser configurations. In short external cavities, regular pulse packages and self-pulsating states can emerge from the interaction between the intrinsic carrier–photon dynamics and the delayed field [23,24,25]. Delayed feedback has also been used to control regenerative pulse trains in semiconductor microcavity lasers, demonstrating that the timing and organization of optical pulses can be manipulated through the feedback dynamics [26]. These studies establish optical feedback as a mechanism capable of generating complex pulsed states; however, a systematic characterization of well-separated regular pulse trains in terms of their existence, repetition rate, pulse width, and duty factor across feedback strength, feedback phase, pump ratio, and relaxation-normalized short delays remains of interest.

Optical feedback is also directly relevant to sensing and metrology. In laser-feedback interferometry and self-mixing sensing, a portion of the emitted field is reflected or scattered back into the laser cavity, where the reinjected field modifies the laser dynamics and carries information about the external target or optical path [27]. Experimental studies of laser-feedback interferometric sensors have shown that the resulting dynamical regime depends jointly on the optical feedback level and the laser bias current [28]. This dependence is particularly relevant to the present work because feedback strength and pump level are also key control parameters governing the formation and repetition rate of the pulse trains. Although the configuration studied here is intended as a controlled short-delay feedback source rather than a self-mixing sensor itself, the ability to identify stable and tunable pulsing regimes is directly relevant to compact optical sources for sensing and ranging systems.

The present study addresses the pulsed semiconductor-laser source rather than a complete sensing system. Accordingly, no direct claim is made regarding system-level sensitivity or dynamic range, which also depend on transmitted optical power, propagation loss, detector response, receiver noise and bandwidth, and signal processing. Nevertheless, the quantities characterized here are directly relevant to pulsed sensing and ranging applications. Pulse width influences the available temporal discrimination, repetition rate determines the measurement update rate and constrains the unambiguous ranging interval, and pulse-amplitude uniformity provides a source-level measure of pulse-to-pulse stability. The maps presented here should therefore be interpreted as a tool for selecting suitable source operating conditions for subsequent sensor-specific design and experimental evaluation.

External-cavity semiconductor lasers (ECSLs) exhibit rich nonlinear dynamics because the delayed optical field interacts coherently with the instantaneous carrier–photon state. Depending on the feedback strength, feedback phase, delay, and pump level, the system can exhibit stable emission, relaxation oscillations, periodic pulsations, regular pulse packages, quasiperiodicity, and chaotic behavior [23,24,29,30,31,32]. In short external cavities, the feedback phase becomes particularly important because small phase variations modify the interference between the intracavity and reinjected fields and can strongly reorganize the resulting dynamics [19,23,33,34].

The short-delay regime considered here is particularly relevant when the feedback delay becomes comparable to the intrinsic relaxation-oscillation period of the solitary laser. In this regime, the delayed optical field returns on the same timescale as the carrier–photon energy exchange, producing a direct interaction between the external-cavity memory and the intrinsic laser dynamics [19,23]. Rather than selecting arbitrary feedback delays, we normalize the delay to the pump-dependent relaxation-oscillation period through the ratio $\tau_{f}/T_{R}$, where $\tau_{f}$ is the optical feedback delay, $T_{R}$ is the relaxation-oscillation period of the solitary laser, and $f_{R}=1/T_{R}$ is the corresponding relaxation-oscillation frequency. Since $f_{R}$ depends on the pump ratio μ, $T_{R}$ is calculated independently for each pump level. Two normalized feedback-delay conditions are investigated: $\tau_{f}/T_{R}=0.5$ and $\tau_{f}/T_{R}=1$. These conditions correspond to reinjection after approximately one-half and one complete intrinsic relaxation-oscillation period, respectively. Because $f_{R}$ increases with the pump level, this normalization provides a physically consistent basis for comparing different pumping conditions. It also connects the simulated feedback delays with millimeter- to centimeter-scale optical paths that are compatible with compact integrated waveguide loops and related feedback structures [18,19].

A central difficulty in mapping pulse generation is distinguishing genuine pulse trains from strongly distorted periodic relaxation oscillations. Peak counting or spectral-harmonic criteria alone can produce false-positive classifications because nonsinusoidal oscillations may exhibit sharp maxima and substantial harmonic content without forming well-separated pulses. In this work, pulse identification is therefore performed directly in the time domain using complementary measures of temporal regularity and pulse shape. The classifier combines significant-peak detection, pulse-to-pulse period regularity, local full width at half maximum, duty factor, valley-to-peak ratio, baseline occupancy, normalized prominence, and autocorrelation. A waveform is classified as a regular pulse train only when it simultaneously exhibits regular recurrence, well-defined narrow maxima, and sufficiently separated low-intensity intervals.

Using this criterion, we investigate deterministic pulse generation in a quadrature-based ECSL model for pump ratios μ=4, 6, 8, and 10, with normalized feedback delays $\tau_{f}/T_{R}=0.5$ and 1. For each of the resulting eight pump–delay conditions, the feedback strength and feedback phase are systematically varied to construct high-resolution maps in the $\left(\mathrm{lg}\left(C\right),\phi_{f}\right)$ plane. The maps identify the regions supporting regular pulse trains and characterize their repetition rate, mean pulse width, and duty factor. In particular, the results reveal a systematic shift toward higher pulse repetition rates as the pump ratio increases, providing a direct mechanism for pump-controlled tuning of the generated pulse trains. The deterministic formulation is intended to establish the underlying pulse-supporting dynamical structure before spontaneous-emission noise, thermal fluctuations, fabrication tolerances, and slow phase drift are introduced.

The principal contributions of this work are summarized as follows:

- We construct high-resolution pulse-generation maps in the feedback-strength and feedback-phase plane for eight pump–delay operating conditions defined by μ=4,6,8,10 and $\tau_{f}/T_{R}=0.5,1$.
- We employ a time-domain pulse-identification procedure that distinguishes well-separated regular pulse trains from strongly distorted periodic oscillations using complementary measures of temporal regularity and pulse shape.
- We characterize the pulse-generation regions through maps of pulse existence, repetition rate, mean pulse width, and duty factor, and demonstrate a systematic increase in repetition rate with increasing pump ratio.
- We relate the normalized short-feedback delays to physically realizable millimeter- to centimeter-scale optical paths compatible with compact and integrated photonic implementations.
- We provide a deterministic reference framework for identifying stable and tunable pulse-generation regimes potentially relevant to compact semiconductor-laser sources for optical sensing, time-of-flight ranging, and integrated photonic systems.

The remainder of this paper is organized as follows. Section 2 introduces the quadrature ECSL model, the pulse-detection and characterization procedure, the selection of the pump-dependent feedback delays, and their connection with integrated photonic implementations. Section 3 presents the pulse-existence, repetition-rate, pulse-width, and duty-factor maps, together with representative temporal traces. Finally, Section 4 summarizes the main conclusions and outlines future work involving stochastic, thermal, and fabrication-related effects.

## 2. Method

The analysis is based on the deterministic ECSL model previously used to study short-delay feedback dynamics [32]. In the present work, however, the objective is not to quantify dynamical instability through Lyapunov exponents, but to identify and characterize operating conditions that produce regular optical pulse trains. Spontaneous-emission and Langevin noise terms are therefore omitted in order to isolate the intrinsic delayed-feedback dynamics, consistently with deterministic analyses of nonlinear semiconductor-laser systems [35,36]. Previous numerical results have shown that noise can introduce pulse-to-pulse timing fluctuations without substantially modifying the mean pulse period [37].

### 2.1. Quadrature ECSL Model

**Figure 1:**
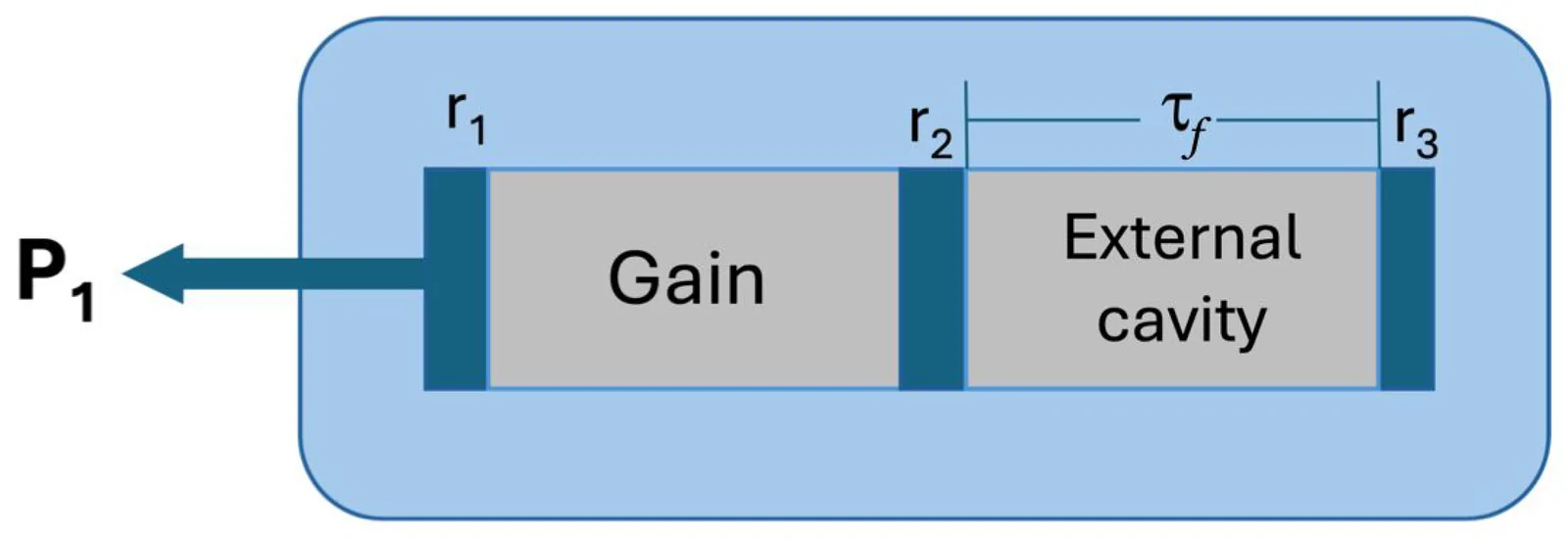
External-cavity semiconductor laser with delayed optical self-feedback. Here, $r_{1}$ is the amplitude reflectivity of the output facet of the laser, $r_{2}$ is the amplitude reflectivity of the laser facet coupled to the external cavity, and $r_{3}$ is the amplitude reflectivity of the external reflector. The feedback delay is denoted by $\tau_{f}$.

For completeness, we summarize the quadrature representation of the short-delay ECSL model used in the simulations; the corresponding self-feedback configuration is shown in Figure 1. The formulation and its numerical properties have been described previously [32,38]; only the definitions required for the pulse-generation analysis are repeated here. The complex field is represented as E(t)=X(t)+iY(t) and is coupled to the carrier variable N(t) through delayed optical self-feedback, where $\tau_{f}$ is the feedback delay, $k_{\mathrm{fb}}$ is the optical-feedback coupling rate, $\eta_{f}$ is the feedback-loop amplitude transmission, and $\phi_{f}$ is the feedback phase.

Define the real gain factor

(1) $$G\left(t\right)=\frac{1}{2}\;g\left(N\left(t\right)-N_{\mathrm{th}}\right),$$

and rotate the delayed field according to

(2) $$\begin{array}{cc} X_{\mathrm{fb}}\left(t\right) & =\cos\phi_{f}\;X\left(t-\tau_{f}\right)-\sin\phi_{f}\;Y\left(t-\tau_{f}\right),\\ Y_{\mathrm{fb}}\left(t\right) & =\sin\phi_{f}\;X\left(t-\tau_{f}\right)+\cos\phi_{f}\;Y\left(t-\tau_{f}\right). \end{array}$$

The quadrature ECSL equations read

(3) $$\left\{\begin{array}{cc} \dot{X}\left(t\right) & =G\left(t\right)\;X\left(t\right)\;-\;\alpha \;G\left(t\right)\;Y\left(t\right)\;+\;k_{\mathrm{fb}}\eta_{f}\;X_{\mathrm{fb}}\left(t\right),\\ \dot{Y}\left(t\right) & =G\left(t\right)\;Y\left(t\right)\;+\;\alpha \;G\left(t\right)\;X\left(t\right)\;+\;k_{\mathrm{fb}}\eta_{f}\;Y_{\mathrm{fb}}\left(t\right),\\ \dot{N}\left(t\right) & =J-\gamma_{N}N\left(t\right)\;-\;\left(\gamma_{P}+g\left(N\left(t\right)-N_{\mathrm{th}}\right)\right)\;\left(X{\left(t\right)}^{2}+Y{\left(t\right)}^{2}\right). \end{array}\right.$$

In Equation (3), X(t) and Y(t) are the in-phase and quadrature components of the complex intracavity optical field E(t), respectively, and N(t) is the carrier population in the active region. The quantity $G\left(t\right)=\frac{1}{2}g\left[N\left(t\right)-N_{\mathrm{th}}\right]$ is the field-gain factor, where *g* is the differential gain coefficient and $N_{\mathrm{th}}$ is the threshold carrier population. The parameter α is the linewidth enhancement factor, *J* is the carrier injection rate, $\gamma_{N}$ is the carrier recombination rate, and $\gamma_{P}$ is the photon decay rate. The parameter $k_{\mathrm{fb}}$ is the optical-feedback coupling rate, $\eta_{f}$ is the feedback-loop amplitude transmission, and $\tau_{f}$ and $\phi_{f}$ are the feedback delay and feedback phase, respectively. The quantities $X_{\mathrm{fb}}\left(t\right)$ and $Y_{\mathrm{fb}}\left(t\right)$ are the in-phase and quadrature components of the delayed optical field after the phase rotation defined in Equation (2). The measured intensity is

(4) $$I\left(t\right)={\left|E\left(t\right)\right|}^{2}=X{\left(t\right)}^{2}+Y{\left(t\right)}^{2}.$$

The subsequent analysis uses the post-transient intensity $I\left(t\right)=X^{2}\left(t\right)+Y^{2}\left(t\right)$ to determine whether each operating point corresponds to a regular pulse train.

### 2.2. Pulse Detection and Characterization Method

The post-transient intensity, $I\left(t\right)=X^{2}\left(t\right)+Y^{2}\left(t\right)$, was analyzed to determine whether each simulated operating point produced a regular train of well-separated optical pulses. For each trace, we define $I_{\min}=\min_{t}I\left(t\right)$, $I_{\max}=\max_{t}I\left(t\right)$, and $\Delta I=I_{\max}-I_{\min}$. Traces with numerically negligible ΔI were directly classified as nonpulsing states.

#### 2.2.1. Peak Detection

Candidate pulses were identified as local maxima satisfying

(5) $$I_{\mathrm{peak}}^{\left(k\right)}\geq I_{\min}+0.35\Delta I,\;P_{k}\geq 0.10\Delta I,$$

where $P_{k}$ is the peak prominence relative to the surrounding valleys. Accepted maxima were additionally required to be separated by at least 20ps to prevent secondary maxima within a single optical pulse from being counted as independent pulses. The accepted peak times are denoted by $t_{p_{k}}$, with $k=1,\ldots ,N_{p}$.

#### 2.2.2. Pulse Metrics and Classification

The temporal intervals between consecutive pulses and the corresponding regularity and repetition-rate measures were calculated as

(6) $$\begin{array}{cc} T_{k} & =t_{p_{k+1}}-t_{p_{k}},\;k=1,\ldots ,N_{p}-1,\\ \;\bar{T} & =\frac{1}{N_{p}-1}\sum_{k=1}^{N_{p}-1}T_{k},\;CV_{T}=\frac{\sigma_{T}}{\bar{T}},\;f_{\mathrm{rep}}=\frac{1}{\bar{T}}, \end{array}$$

where $\sigma_{T}$ is the standard deviation of the consecutive pulse periods.

Pulse width was evaluated relative to a local baseline to avoid misclassifying the narrow upper portions of strongly distorted relaxation oscillations. For the *k*-th pulse, the local baseline and half-maximum level were defined from the neighboring valleys as

(7) $$\begin{array}{cc} I_{\mathrm{base}}^{\left(k\right)} & =\frac{I_{\min,L}^{\left(k\right)}+I_{\min,R}^{\left(k\right)}}{2},\\ I_{1/2}^{\left(k\right)} & =I_{\mathrm{base}}^{\left(k\right)}+\frac{I_{\mathrm{peak}}^{\left(k\right)}-I_{\mathrm{base}}^{\left(k\right)}}{2},\\ W_{k} & =t_{\mathrm{right}}^{\left(k\right)}-t_{\mathrm{left}}^{\left(k\right)},\\ \bar{W} & =\frac{1}{N_{W}}\sum_{k=1}^{N_{W}}W_{k},\;D_{\mathrm{local}}=\frac{\bar{W}}{\bar{T}}, \end{array}$$

where the half-maximum crossings $t_{\mathrm{left}}^{\left(k\right)}$ and $t_{\mathrm{right}}^{\left(k\right)}$ were obtained by linear interpolation, and $N_{W}$ is the number of finite, positive pulse widths.

Pulse separation and prominence were quantified through

(8) $$\begin{array}{cc} R_{\mathrm{VP}}^{\left(k\right)} & =\frac{I_{\mathrm{base}}^{\left(k\right)}}{I_{\mathrm{peak}}^{\left(k\right)}},\;{\bar{R}}_{\mathrm{VP}}=\frac{1}{N_{W}}\sum_{k=1}^{N_{W}}R_{\mathrm{VP}}^{\left(k\right)},\\ \;P_{\mathrm{norm}}^{\left(k\right)} & =\frac{I_{\mathrm{peak}}^{\left(k\right)}-I_{\mathrm{base}}^{\left(k\right)}}{\Delta I},\;{\bar{P}}_{\mathrm{norm}}=\frac{1}{N_{W}}\sum_{k=1}^{N_{W}}P_{\mathrm{norm}}^{\left(k\right)}. \end{array}$$

Low values of ${\bar{R}}_{\mathrm{VP}}$ indicate deep inter-pulse valleys, whereas large ${\bar{P}}_{\mathrm{norm}}$ values indicate well-defined pulse maxima.

To ensure that the waveform spends a significant fraction of time near its low-intensity state, the baseline-occupancy fraction was calculated as

(9) $$\begin{array}{cc} I_{\mathrm{low}} & =I_{\min}+0.20\Delta I,\\ F_{\mathrm{base}} & =\frac{1}{N_{s}}\sum_{j=1}^{N_{s}}H\left(I_{\mathrm{low}}-I_{j}\right), \end{array}$$

where $N_{s}$ is the number of temporal samples and H(·) is the Heaviside indicator function.

Temporal recurrence was independently evaluated using the normalized autocorrelation

(10) $$\begin{array}{cc} \tilde{I}\left(t\right) & =I(t)-\langle I(t)\rangle ,\\ R_{II}\left(\tau \right) & =\frac{\langle \tilde{I}\left(t\right)\tilde{I}\left(t+\tau \right)\rangle }{\langle {\tilde{I}}^{\;2}\left(t\right)\rangle }. \end{array}$$

The first nonzero-lag local maximum of $R_{II}\left(\tau \right)$ was denoted by $R_{\mathrm{AC}}$.

A trace was classified as a regular pulse train only when both the periodicity and pulse-shape conditions were satisfied:

(11) $$\begin{array}{cc} C_{\mathrm{periodic}}= & \left(N_{p}\geq 5\right)\wedge \left(CV_{T}\leq 0.05\right)\wedge \left(R_{\mathrm{AC}}\geq 0.80\right),\\ C_{\mathrm{shape}}= & \left(D_{\mathrm{local}}\leq 0.25\right)\wedge \left({\bar{R}}_{\mathrm{VP}}\leq 0.20\right)\\ \; & \wedge \left(F_{\mathrm{base}}\geq 0.45\right)\wedge \left({\bar{P}}_{\mathrm{norm}}\geq 0.60\right). \end{array}$$

Accordingly, PulseClass=1 when $C_{\mathrm{periodic}}\wedge C_{\mathrm{shape}}$ is satisfied and PulseClass=0 otherwise. The combined criteria reject periodic but continuously modulated waveforms that do not exhibit well-separated optical pulses.

#### 2.2.3. Pulse-Amplitude Uniformity

For traces classified as pulse trains, pulse-to-pulse amplitude uniformity was evaluated relative to the local baseline. The local pulse amplitude, its mean value, and its coefficient of variation were calculated as

(12) $$\begin{array}{cc} A_{k} & =I_{\mathrm{peak}}^{\left(k\right)}-I_{\mathrm{base}}^{\left(k\right)},\\ \bar{A} & =\frac{1}{N_{A}}\sum_{k=1}^{N_{A}}A_{k},\\ CV_{A} & =\frac{\sigma_{A}}{\bar{A}}, \end{array}$$

where $N_{A}$ is the number of finite positive pulse amplitudes and $\sigma_{A}$ is their standard deviation. The final three-state classification was

(13) $$\mathrm{PulseUniformityClass}=\left\{\begin{array}{cc} 0, & \mathrm{PulseClass}=0,\\ 1, & \mathrm{PulseClass}=1\;\wedge \;CV_{A}>0.05,\\ 2, & \mathrm{PulseClass}=1\;\wedge \;CV_{A}\leq 0.05. \end{array}\right.$$

Thus, classes 0, 1, and 2 correspond to nonpulsing states, nonuniform pulse trains, and amplitude-uniform pulse trains, respectively. The uniformity criterion is applied only after a waveform has satisfied the regular pulse-train criteria.

For each pulsing operating point, the stored quantities were $N_{p}$, $\bar{T}$, $f_{\mathrm{rep}}$, $CV_{T}$, $\bar{W}$, $D_{\mathrm{local}}$, ${\bar{R}}_{\mathrm{VP}}$, $F_{\mathrm{base}}$, ${\bar{P}}_{\mathrm{norm}}$, $R_{\mathrm{AC}}$, $\bar{A}$, and $CV_{A}$. These metrics were subsequently used to construct the pulse-existence, repetition-rate, pulse-width, duty-factor, and amplitude-uniformity maps.

### 2.3. Selection of the Feedback Delay

The feedback delay was normalized to the pump-dependent relaxation-oscillation period of the solitary laser. Using the small-signal approximation,

$$f_{R}\left(\mu \right)=\frac{1}{2\pi }\sqrt{g\left(\mu J_{\mathrm{th}}-\gamma_{N}N_{\mathrm{th}}\right)},$$

with $T_{R}\left(\mu \right)=1/f_{R}\left(\mu \right)$. For each pump ratio, two feedback conditions were investigated: $\tau_{f}=T_{R}$ and $\tau_{f}=0.5T_{R}$. The corresponding relaxation-oscillation frequencies are summarized in Table 1.

These frequencies define the pump-dependent relaxation-oscillation periods used to select the two normalized feedback-delay conditions, $\tau_{f}/T_{R}=1$ and 0.5. The corresponding physical delay values are reported in Table 2.

### 2.4. Connection with Integrated Photonic Implementations

The simulated feedback delays can be related to effective optical path lengths through $L_{\mathrm{eff}}=\frac{c\tau_{f}}{n_{g}},$ where $n_{g}$ is the group index and $L_{\mathrm{eff}}$ is the total effective propagation length associated with the delayed feedback path. Table 2 gives representative values for silicon, SiN-like, and InP/SOI-like platforms.

The resulting effective path lengths range from a few millimeters to several centimeters. The shorter condition, $\tau_{f}=0.5T_{R}$, reduces the required feedback-path length by approximately one half relative to $\tau_{f}=T_{R}$, making the higher-pump operating conditions particularly compatible with compact folded or spiral waveguide implementations.

The conversion from feedback delay to effective propagation length should be interpreted as a first-order geometrical feasibility estimate rather than as a complete integrated-device design. In a practical implementation, the operating regime is determined not only by the feedback delay but also by the amplitude and phase of the reinjected field. Propagation, bending, and coupling losses, together with the effective reflectivity of the feedback structure, determine the returned optical-field amplitude and therefore the effective feedback strength *C*. Similarly, the accumulated optical phase, thermal drift, and fabrication-induced variations affect the feedback phase $\phi_{f}$. Consequently, integrated implementations with the same nominal delay may correspond to different points in the $\left(\mathrm{lg}\left(C\right),\phi_{f}\right)$ plane unless both the feedback amplitude and phase are appropriately controlled. The path lengths reported in Table 2 therefore establish that the delays considered here are compatible with compact photonic circuits, while practical realization requires platform-specific control of feedback loss and phase.

The feedback coordinate used throughout the maps is $C=K\tau_{p}$, where $K=k_{\mathrm{fb}}\eta_{f}$ is the effective feedback rate and $\tau_{p}$ is the photon lifetime. The detailed relation between this normalized parameter and physical quantities such as facet reflectivities, external-reflector strength, and feedback-path losses has been discussed previously for the same ECSL formulation [32]. In the present work, *C* is used as the control parameter for identifying pulse-generation regimes.

In this formulation, the mirror reflectivities are incorporated through effective lumped parameters rather than treated as independent variables. The laser-facet reflectivities contribute to the photon decay rate $\gamma_{P}=1/\tau_{p}$ and consequently to the reference threshold quantities $N_{\mathrm{th}}$ and $J_{\mathrm{th}}$. For a simple facet–mirror feedback configuration, the feedback rate $k_{\mathrm{fb}}$ incorporates the coupling through the feedback-side laser facet and the reflectivity of the external reflector, whereas $\eta_{f}$ represents the amplitude transmission of the external feedback path, including propagation and coupling losses. Thus, $C=k_{\mathrm{fb}}\eta_{f}\tau_{p}$ provides the effective feedback strength used in the present maps. The facet and mirror reflectivities are not varied independently in this study; instead, their effects are contained in the fixed laser parameters and in the swept effective feedback strength *C* [32].

### 2.5. Simulation Parameters

All simulations used the same constant deterministic history, $X\left(t\right)=A_{\mathrm{ss}}$, Y(t)=0, and $N\left(t\right)=N_{\mathrm{th}}$ for $t\in [-\tau_{f},0]$, where $A_{\mathrm{ss}}=\sqrt{\left(J-\gamma_{N}N_{\mathrm{th}}\right)/\gamma_{P}}$. The DDEs were integrated using a fourth-order Runge–Kutta method with linear interpolation of the delayed states. For each pump ratio, the feedback delay was selected as either $\tau_{f}=T_{R}$ or $\tau_{f}=0.5T_{R}$, with $T_{R}$ determined from the corresponding pump-dependent relaxation-oscillation period reported in Table 1. The fixed ECSL parameters are listed in Table 3, while the numerical settings and parameter-sweep ranges are summarized in Table 4.

The fixed values in Table 3 were selected to represent a typical single-mode semiconductor laser operating near 1.55 μm and are consistent with representative values reported in the literature [39,40]. The same parameter set was used in our previous short-delay ECSL study, allowing direct comparison with the present pulse-generation analysis. These quantities were kept fixed in order to isolate the influence of the parameters explicitly explored in the maps: pump ratio, feedback strength, feedback phase, and normalized feedback delay. The exact locations and widths of the pulsing regions are expected to depend quantitatively on the fixed laser parameters. For example, *g*, α, $\gamma_{N}$, and $\gamma_{P}$ modify the intrinsic carrier–photon dynamics and therefore can shift the pulse-generation boundaries and repetition frequencies. The use of the normalized delay $\tau_{f}/T_{R}$ partially compensates for changes in the intrinsic timescale by relating the external delay to the corresponding relaxation-oscillation period. Consequently, the reported maps should be interpreted as representative of this parameter set rather than as universal boundaries for all semiconductor lasers.

The nominal sweep extends to C=1; however, the maps report only the numerically convergent portion of the parameter space, extending to approximately lg(C)=−0.3 (C≈0.5).

## 3. Results

**Figure 2:**
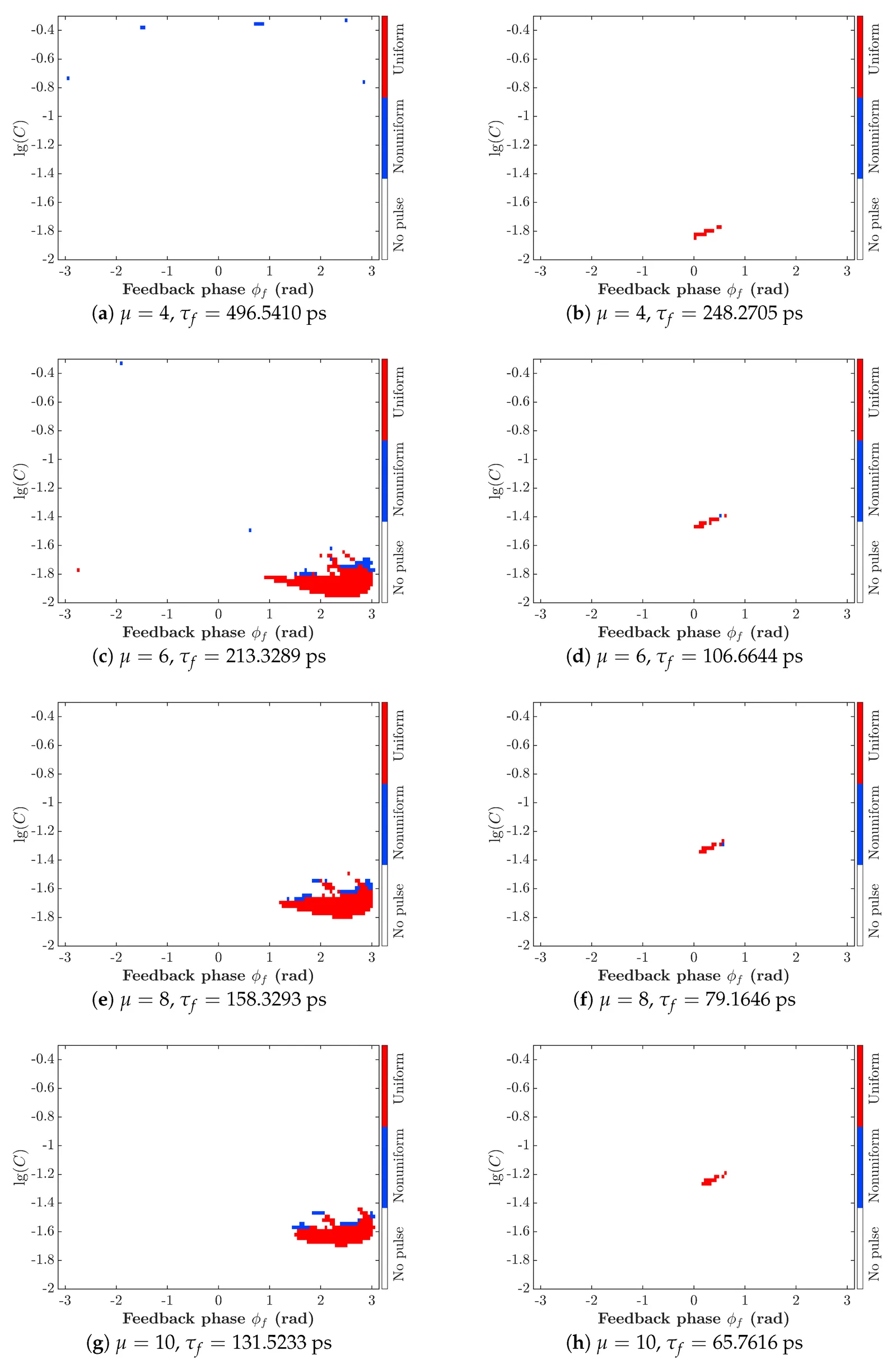
Pulse-generation maps as a function of feedback phase $\phi_{f}$ and feedback strength *C*. Rows correspond to μ=4, 6, 8, and 10, while the left and right columns correspond to $\tau_{f}=T_{R}$ and $\tau_{f}=0.5T_{R}$, respectively. White denotes nonpulsing operation, blue denotes regular pulse trains with nonuniform pulse amplitudes, and red denotes amplitude-uniform pulse trains satisfying $CV_{A}\leq 0.05$. Each colored cell represents an individually simulated operating point in the discrete $\left(\mathrm{lg}\left(C\right),\phi_{f}\right)$ parameter grid; therefore, the visible grid structure reflects the numerical sampling rather than the graphical resolution of the figure.

Figure 2 shows the pulse-generation maps in the $\left(\mathrm{lg}\left(C\right),\phi_{f}\right)$ plane for the eight pump–delay combinations. White regions denote nonpulsing states, blue regions correspond to nonuniform pulse trains, and red regions indicate amplitude-uniform pulse trains satisfying $CV_{A}\leq 0.05$. Both red and blue regions therefore represent valid pulse trains; the color distinction refers only to pulse-to-pulse amplitude uniformity.

At μ=4, pulsing remains sparse for both normalized delays. A substantial expansion of the pulse-generation region appears for μ=6, 8, and 10, particularly when the feedback delay is matched to the pump-dependent relaxation-oscillation period, $\tau_{f}=T_{R}$. In these cases, extended pulsing domains are concentrated mainly at positive feedback phases and weak-to-moderate feedback strengths, with uniform pulse trains occupying a large fraction of the detected regions. In contrast, the corresponding $\tau_{f}=0.5T_{R}$ cases generally produce much smaller and more isolated pulsing islands.

These results show that the normalized delay ratio $\tau_{f}/T_{R}$ strongly organizes the formation of regular pulse trains. For μ=6, 8, and 10, matching the feedback delay to $T_{R}$ substantially enlarges the accessible pulsing region, whereas at μ=4, pulse generation remains limited for both delays. The amplitude-uniformity classification further distinguishes stable, nearly constant-amplitude pulse trains from valid pulsing regimes exhibiting pulse-to-pulse amplitude modulation.

**Figure 3:**
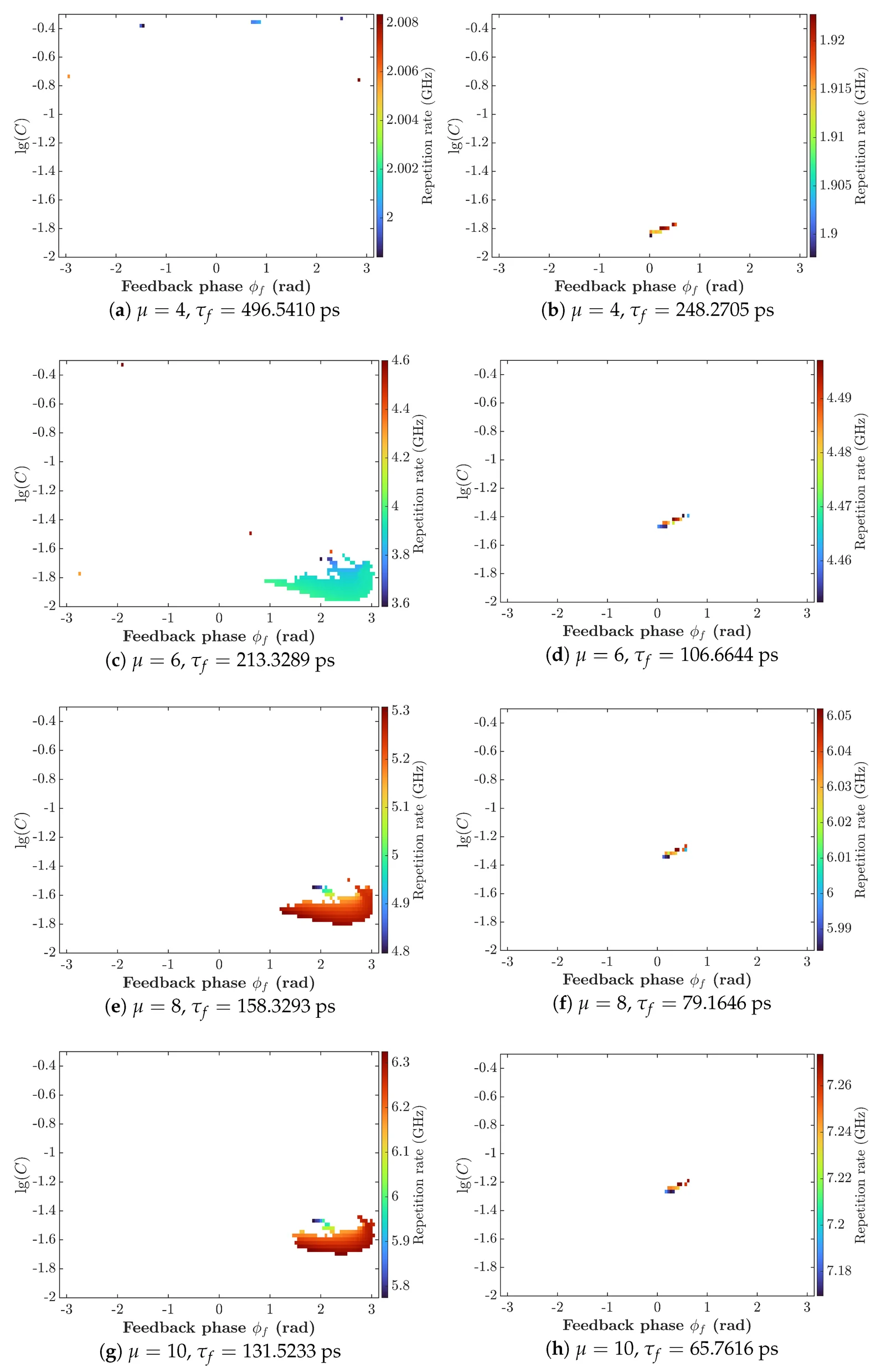
Pulse repetition-rate maps for the operating points classified as regular pulse trains. Rows correspond to μ=4, 6, 8, and 10, and the left and right columns correspond to $\tau_{f}=T_{R}$ and $\tau_{f}=0.5T_{R}$, respectively. The color scale gives the repetition rate $f_{\mathrm{rep}}=1/T$ in GHz. White regions correspond to operating points that do not satisfy the pulse-train classification criteria.

**Figure 4:**
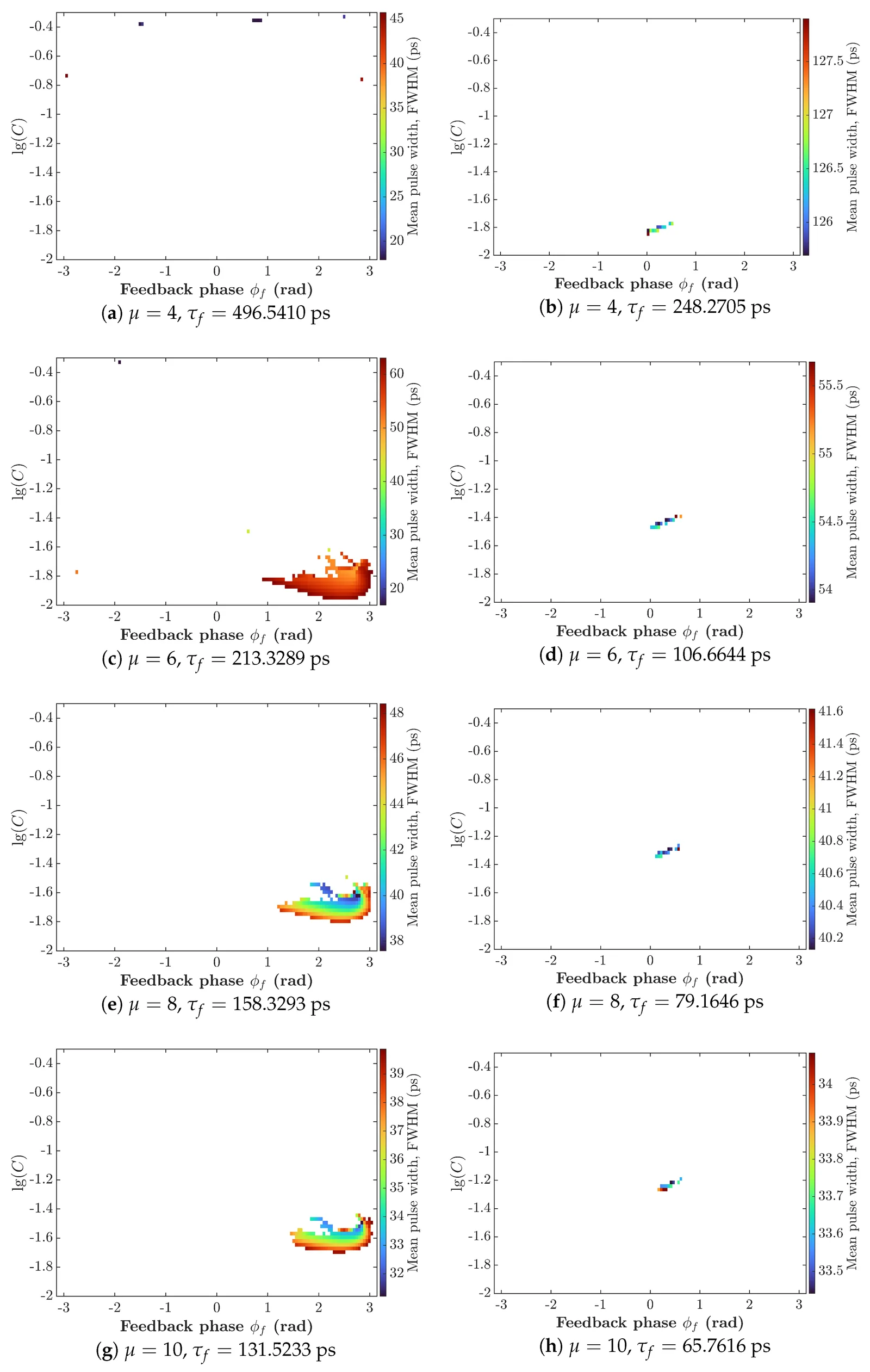
Mean pulse-width maps for the operating points classified as regular pulse trains. Rows correspond to μ=4, 6, 8, and 10, and the left and right columns correspond to $\tau_{f}=T_{R}$ and $\tau_{f}=0.5T_{R}$, respectively. The color scale gives the mean local full width at half maximum *W* in ps. White regions correspond to operating points that do not satisfy the pulse-train classification criteria.

**Figure 5:**
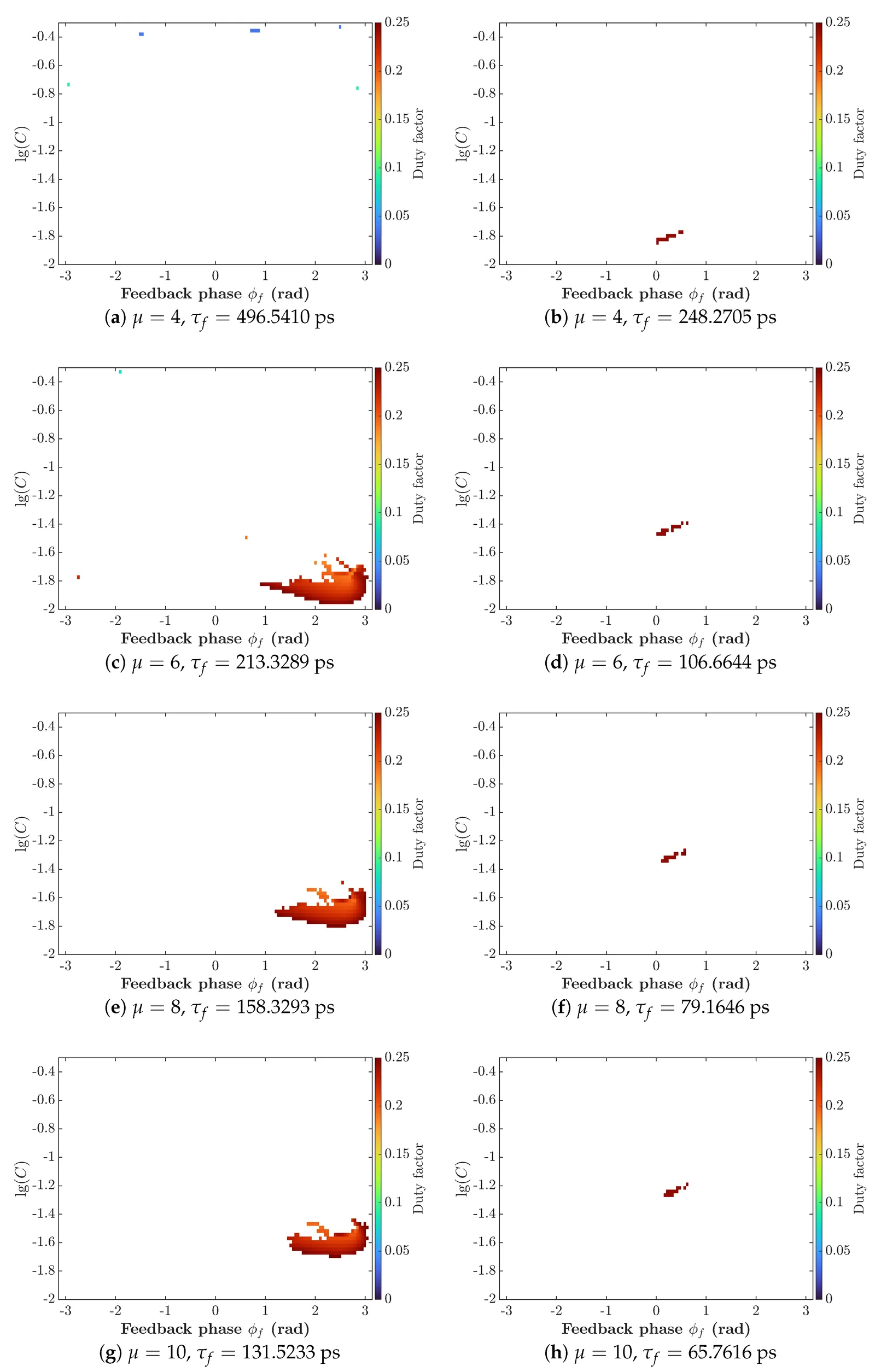
Duty-factor maps for the operating points classified as regular pulse trains. Rows correspond to μ=4, 6, 8, and 10, and the left and right columns correspond to $\tau_{f}=T_{R}$ and $\tau_{f}=0.5T_{R}$, respectively. The color scale gives the local duty factor $D_{\mathrm{local}}=W/T$, representing the fraction of each repetition period occupied by the pulse. White regions correspond to operating points that do not satisfy the pulse-train classification criteria.

Figure 2, Figure 3, Figure 4 and Figure 5 use the same panel organization to facilitate direct comparison among operating conditions. From top to bottom, the rows correspond to pump ratios μ=4, 6, 8, and 10, respectively. For each pump ratio, the left panel corresponds to $\tau_{f}=T_{R}$ and the right panel to $\tau_{f}=0.5T_{R}$. In all maps, the horizontal coordinate is the feedback phase $\phi_{f}$ and the vertical coordinate is the decimal logarithm of the dimensionless feedback strength, lg(C). Each map therefore shows how the corresponding pulse characteristic changes over the same feedback-strength–feedback-phase parameter space.

Table 5 summarizes the number of detected pulsing states and their principal temporal characteristics for the eight pump–delay combinations.

The characteristic feedback timescales considered here are also comparable to those explored experimentally in semiconductor lasers operating in the short-cavity regime. Heil et al. experimentally reported regular pulse-package dynamics for an external-cavity length of approximately 3.7 cm, corresponding to an external-cavity round-trip frequency of about 4.1 GHz and hence to a round-trip delay of approximately 244 ps [33]. This delay lies within the 213–248 ps range considered in the present simulations. In the experiments of Heil et al., the regular pulse packages consisted of a slower envelope containing fast intensity pulsations separated by approximately the external-cavity round-trip time. Thus, the comparison with the present results concerns the characteristic short-feedback timescale rather than an identification of the two pulsing regimes. Experiments on an integrated semiconductor laser with optical feedback have also shown that increasing the injection current, and hence the relaxation-oscillation frequency, changes the dynamical regime relative to the external-cavity frequency [19]. This experimental observation is consistent with the present use of the normalized delay $\tau_{f}/T_{R}$ to compare feedback dynamics at different pump levels. Because the experimental devices and laser parameters differ from those used here, these comparisons should be regarded as agreement in characteristic timescales and qualitative dynamical trends, rather than as a point-by-point quantitative validation.

To assess the sensitivity of the pulse-generation results to the initial history, the complete $\left(\mathrm{lg}\left(C\right),\phi_{f}\right)$ sweep was repeated for the representative condition μ=8 and $\tau_{f}$ = $T_{R}$ = 158.3293 ps using four additional constant histories. The initial optical-field amplitude and carrier population were independently perturbed by ±10% relative to the nominal history. As summarized in Table 6, the nominal case produced 228 pulsing points, whereas the perturbed histories produced between 230 and 237 pulsing points, corresponding to variations below 4%. The numbers of uniform and nonuniform pulse trains and the $D_{\mathrm{local}}$ ranges were also similar. One field-amplitude perturbation produced an extended maximum repetition rate of 5.97 GHz, indicating some local sensitivity of individual operating points. Overall, however, the tested perturbations do not indicate a strong dependence of the pulse-generation statistics on the initial history for this representative operating condition. A comprehensive multistability analysis over all pump–delay combinations remains beyond the scope of the present study.

$$\begin{array}{cccc} H_{1}: & X\left(t\right)=0.9A_{\mathrm{ss}}, & Y(t)=0, & N\left(t\right)=N_{\mathrm{th}},\\ H_{2}: & X\left(t\right)=1.1A_{\mathrm{ss}}, & Y(t)=0, & N\left(t\right)=N_{\mathrm{th}},\\ H_{3}: & X\left(t\right)=A_{\mathrm{ss}}, & Y(t)=0, & N\left(t\right)=0.9N_{\mathrm{th}},\\ H_{4}: & X\left(t\right)=A_{\mathrm{ss}}, & Y(t)=0, & N\left(t\right)=1.1N_{\mathrm{th}}. \end{array}$$

Figure 3 shows the repetition-rate maps for the operating points classified as pulse trains, with $f_{\mathrm{rep}}=1/\bar{T}$. White regions denote nonpulsing states, whereas the colored regions indicate the repetition frequency of the detected pulse trains.

The repetition rate increases systematically with pump ratio. At μ=4, the sparse pulsing regions exhibit frequencies near 1.9–2.0GHz. For μ=6, the extended $\tau_{f}=T_{R}$ region spans approximately 3.6–4.6GHz, while the corresponding half-period case is confined to a small island near 4.5GHz. At μ=8, the main pulsing domain reaches approximately 4.8–5.3GHz, whereas the $\tau_{f}=0.5T_{R}$ case produces repetition rates close to 6GHz. For μ=10, the full-period case covers approximately 5.77–6.33GHz, while the shorter-delay island reaches 7.17–7.27GHz.

Overall, Figure 3 shows that increasing μ shifts the pulse-generation regimes toward higher repetition rates, consistent with the corresponding increase in the relaxation-oscillation frequency. At the same time, $\tau_{f}=T_{R}$ generally provides broader accessible pulsing regions for μ=6, 8, and 10, whereas $\tau_{f}=0.5T_{R}$ produces smaller and more isolated domains. Thus, the feedback parameters control not only the existence of pulsed operation but also the attainable repetition-frequency range.

Figure 4 shows the mean pulse-width maps for the operating points classified as regular pulse trains. The reported width, $\bar{W}$, corresponds to the mean local full width at half maximum. White regions denote operating points that do not satisfy the pulse-train classification criteria, whereas the colored regions indicate the mean pulse width of the detected pulse trains.

The pulse width generally decreases as the pump ratio increases. For μ=4, the detected widths range from approximately 20 to 45ps for $\tau_{f}=T_{R}$, while the small $\tau_{f}=0.5T_{R}$ island produces much broader pulses near 126–128ps. At μ=6, the full-period case spans approximately 20–60ps, whereas the half-period case is restricted to about 54–56ps. For μ=8, the corresponding ranges are approximately 38–48ps and 40–42ps, while for μ=10 they decrease to approximately 32–39ps and 33.5–34.1ps, respectively.

Overall, Figure 4 shows that increasing μ shifts the pulse-generation regimes toward shorter temporal widths, consistent with the reduction in the characteristic relaxation-oscillation timescale. The $\tau_{f}=T_{R}$ cases generally provide broader accessible pulsing regions, whereas $\tau_{f}=0.5T_{R}$ produces smaller islands with more narrowly distributed pulse widths. In particular, the μ=8 and μ=10 full-period cases support tens-of-picoseconds pulses over extended regions of parameter space.

Figure 5 shows the duty-factor maps for the operating points classified as regular pulse trains. The local duty factor is defined as $D_{\mathrm{local}}=\bar{W}/\bar{T}$ and represents the fraction of each repetition period occupied by the pulse. White regions denote nonpulsing states, whereas the colored regions indicate the corresponding duty factor.

For μ=4, the few detected pulsing points exhibit duty factors below approximately 0.10 for $\tau_{f}=T_{R}$, while the small $\tau_{f}=0.5T_{R}$ island reaches values near 0.24. At μ=6, the full-period case spans approximately 0.078–0.250, whereas the half-period case is confined to 0.242–0.248. For μ=8, the corresponding ranges are 0.191–0.250 and 0.243–0.250, respectively, while for μ=10 they are 0.193–0.250 and 0.243–0.245.

Overall, Figure 5 shows that all detected pulse trains satisfy $D_{\mathrm{local}}\leq 0.25$. The $\tau_{f}=T_{R}$ cases generally provide larger pulsing regions and a wider range of temporal occupancies, whereas $\tau_{f}=0.5T_{R}$ produces smaller islands with duty factors concentrated near the classification threshold. Low values of $D_{\mathrm{local}}$ identify operating points with better-separated pulses and longer low-intensity intervals between consecutive emissions.

**Figure 6:**
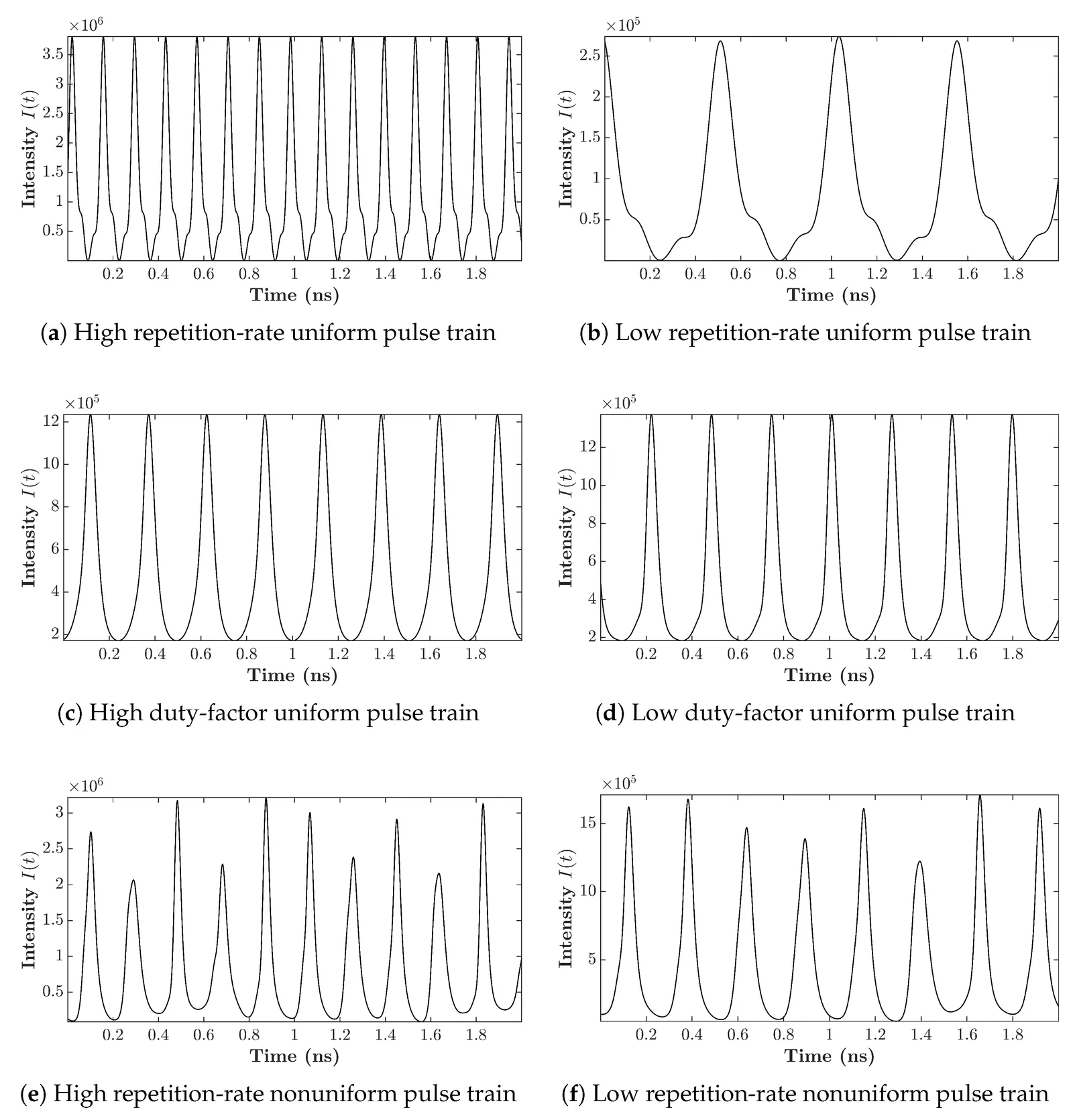
Representative post-transient intensity traces I(t) illustrating the main pulse-train behaviors identified in this work. Panels (**a**,**b**) show uniform pulse trains with high and low repetition rates, respectively. Panels (**c**,**d**) show uniform pulse trains with high and low duty factor, respectively, highlighting the difference in the fraction of each period occupied by the pulse. Panels (**e**,**f**) show nonuniform pulse trains with high and low repetition rates, respectively. These examples illustrate the diversity of temporal waveforms captured by the pulse-classification procedure and provide a direct visual interpretation of the pulse-existence, repetition-rate, and duty-factor maps presented in this paper.

Figure 6 shows representative post-transient intensity traces selected from the pulse-generation maps over a 2ns observation window. The examples illustrate uniform pulse trains with different repetition rates and duty factors, as well as nonuniform pulse trains. The corresponding operating parameters and pulse metrics are summarized in Table 7.

Figure 6a,b illustrate the pump-controlled variation in the repetition rate, ranging from approximately 7.27GHz at μ=10 to 1.92GHz at μ=4, while maintaining nearly uniform pulse amplitudes. Figure 6c,d compare uniform pulse trains with different duty factors for the same pump ratio and feedback delay, illustrating the variation in pulse duration relative to the repetition period.

Figure 6e,f show valid but nonuniform pulse trains with noticeable pulse-to-pulse amplitude variations. Together, the six traces provide direct time-domain confirmation of the pulse-existence, repetition-rate, duty-factor, and amplitude-uniformity information encoded in the corresponding parameter-space maps.

## 4. Conclusions

In this work, deterministic pulse generation in external-cavity semiconductor lasers with short-delay optical self-feedback was investigated using a quadrature-based delay-differential model. High-resolution maps in the $\left(\mathrm{lg}\left(C\right),\phi_{f}\right)$ plane were constructed for pump ratios μ=4,6,8, and 10, with normalized feedback delays $\tau_{f}/T_{R}=0.5$ and 1. The analysis identifies the operating regions that support regular pulse trains and characterizes their repetition rate, mean pulse width, duty factor, and pulse-amplitude uniformity.

For μ=6, 8, and 10, the most extended pulse-generation regions were obtained for $\tau_{f}=T_{R}$, whereas $\tau_{f}=0.5T_{R}$ generally produced smaller and more isolated pulsing domains. At μ=4, pulsing remained sparse for both delays. Increasing the pump ratio shifted the pulsing regimes toward higher repetition rates, from approximately 2GHz at μ=4 to above 7GHz at μ=10, while the higher-pump cases produced pulse widths in the tens-of-picoseconds range. These results show that pump level, feedback phase, feedback strength, and normalized delay jointly determine the accessible pulsing regimes.

A time-domain classifier was used to distinguish well-separated pulse trains from strongly distorted periodic oscillations by combining temporal regularity and pulse-shape criteria. An additional amplitude-uniformity criterion separated valid pulsing states into nearly uniform and nonuniform pulse trains. The resulting maps therefore provide more than a binary identification of pulsed operation and allow operating regions to be selected according to repetition rate, pulse width, duty factor, and amplitude stability.

The simulated delays correspond to effective optical feedback paths ranging from a few millimeters to several centimeters, depending on pump level, normalized delay, and waveguide group index. These dimensions are compatible with folded or spiral feedback structures in silicon, SiN-like, and InP/SOI-like photonic platforms. The short-delay self-feedback configuration is therefore potentially attractive for compact pulse sources for optical sensing and ranging, microwave photonics, timing applications, and integrated photonic systems without requiring an independent master laser or high-frequency electrical modulation.

The present results correspond to ideal deterministic conditions. Future work should assess the robustness of the identified pulsing regions in the presence of spontaneous-emission and Langevin noise, thermal detuning, gain compression, parameter drift, propagation loss, and fabrication tolerances, as well as possible multistability associated with different initial histories. Experimental validation will ultimately be required to determine the practical stability of the predicted operating regions. Overall, the results show that pump-dependent short-delay optical feedback provides a compact and configurable mechanism for generating and controlling regular pulse trains in external-cavity semiconductor lasers.

## Figures and Tables

**Table 1:** Relaxation-oscillation frequency for the pump ratios considered in the pulse-generation simulations.

μ	$f_{R}$ (GHz)
4	2.013932241
6	4.687598450
8	6.315950870
10	7.603215869

**Table 2:** Representative effective feedback-path lengths associated with the delays used in the pulse-generation simulations.

μ	$\tau_{f}/T_{R}$	$\tau_{f}$	Silicon	SiN-like	InP/SOI-like
		(ps)	$n_{g}\approx 4.0$	$n_{g}\approx 2.0$	$n_{g}\approx 3.6$
4	1.0	496.5410	37.2 mm	74.4 mm	41.3 mm
4	0.5	248.2705	18.6 mm	37.2 mm	20.7 mm
6	1.0	213.3289	16.0 mm	32.0 mm	17.8 mm
6	0.5	106.6644	8.0 mm	16.0 mm	8.9 mm
8	1.0	158.3293	11.9 mm	23.7 mm	13.2 mm
8	0.5	79.1646	5.9 mm	11.9 mm	6.6 mm
10	1.0	131.5233	9.9 mm	19.7 mm	11.0 mm
10	0.5	65.7616	4.9 mm	9.9 mm	5.5 mm

**Table 3:** Fixed ECSL parameters used in the simulations. The same parameter set was employed in our previous short-delay study [32] and is consistent with representative semiconductor-laser parameters reported in the literature [39,40].

Parameter	Symbol	Value
Differential gain coefficient	*g*	$5.67\times 10^{3}\;s^{-1}$
Threshold carrier number	$N_{\mathrm{th}}$	$2.214\times 10^{8}$
Linewidth enhancement factor	α	3
Threshold injection rate	$J_{\mathrm{th}}$	$6.24\times 10^{16}\;s^{-1}$
Carrier recombination rate	$\gamma_{N}$	$1.0\times 10^{9}\;s^{-1}$
Photon decay rate/lifetime	$\gamma_{P}$, $\tau_{p}=1/\gamma_{P}$	$3.33\times 10^{11}\;s^{-1}$, 3.0ps

**Table 4:** Simulation and parameter-sweep settings used to generate the ECSL pulse maps.

Parameter	Symbol/Definition	Value
Pump ratio	$\mu =J/J_{\mathrm{th}}$	μ∈{4,6,8,10}
Feedback-loop amplitude transmission	$\eta_{f}$	0.9
Dimensionless feedback strength	$C=k_{\mathrm{fb}}\eta_{f}\tau_{p}$	$N_{C}=80$ logarithmically spaced values over $C\in [10^{-2},1]$
Feedback phase	$\phi_{f}$	$N_{\phi }=128$ uniformly spaced values over $\phi_{f}\in \left[-\pi ,\pi \right]$
Feedback delay	$\tau_{f}$	$\tau_{f}/T_{R}\in \left\{0.5,1.0\right\}$
Simulation time step	Δt	Approximately 1ps
Total simulation time	$T_{\mathrm{sim}}$	200ns
Discarded transient	$t_{\mathrm{trans}}$	50ns
Analyzed window	$T_{\mathrm{post}}$	150ns

**Table 5:** Summary of the pulse-generation regions obtained for the eight pump–delay combinations.

μ	$\tau_{f}$ (ps)	Pulse Points	Uniform Points	Nonuniform Points	$f_{\mathrm{rep}}$ (GHz)	$D_{\mathrm{local}}$ Range
4	496.5410	9	0	9	2.00–2.01	0.036–0.092
4	248.2705	12	12	0	1.90–1.92	0.241–0.243
6	213.3289	262	227	35	3.59–4.60	0.078–0.250
6	106.6644	14	13	1	4.45–4.50	0.242–0.248
8	158.3293	228	199	29	4.80–5.31	0.191–0.250
8	79.1646	13	12	1	5.98–6.05	0.243–0.250
10	131.5233	191	166	25	5.77–6.33	0.193–0.250
10	65.7616	13	13	0	7.17–7.27	0.243–0.245

**Table 6:** Sensitivity of the pulse-generation results to the initial history for the representative condition μ=8 and $\tau_{f}=T_{R}=158.3293$ ps.

History	Pulse Points	Uniform	Nonuniform	$f_{\mathrm{rep}}$ (GHz)	$D_{\mathrm{local}}$ Range
$H_{0}$	228	199	29	4.80–5.31	0.191–0.250
$H_{1}$	232	201	31	4.80–5.97	0.190–0.250
$H_{2}$	230	197	33	4.80–5.31	0.188–0.250
$H_{3}$	231	201	30	4.80–5.30	0.190–0.250
$H_{4}$	237	204	33	4.80–5.30	0.188–0.250

**Table 7:** Operating points and pulse metrics for the representative traces in Figure 6.

Panel	μ	$\tau_{f}$ (ps)	lg(C)	$\phi_{f}$ (rad)	$f_{\mathrm{rep}}$ (GHz)	$D_{\mathrm{local}}$
(a)	10	65.7616	−1.215	0.421	7.273	0.243
(b)	4	248.2705	−1.810	0.272	1.919	0.242
(c)	6	213.3289	−1.848	1.707	3.941	0.227
(d)	6	213.3289	−1.722	2.301	3.809	0.193
(e)	8	158.3293	−1.544	2.894	5.185	0.221
(f)	6	213.3289	−1.722	2.894	3.903	0.228

## Data Availability

Data underlying the results presented in this paper are not publicly available at this time but may be obtained from the authors upon reasonable request.
